# Optical excited states of archetypical blue light emitter, dimethyl-acridine diphenyl-sulfone derivative (DMAC-DPS) in solid films, studied by electroabsorption spectroscopy

**DOI:** 10.1038/s41598-025-29261-2

**Published:** 2025-11-21

**Authors:** Daniel Pelczarski, Malgorzata Makowska-Janusik, Waldemar Stampor

**Affiliations:** 1 Department of Molecular Photophysics, Institute of Applied Physics and Mathematics, Gdańsk University of Technology, 11/12 Narutowicza str, 80-233 Gdańsk, Poland; 2 Faculty of Science and Technology, Jan Dlugosz University, Al. Armii Krajowej 13/15, 42-200 Częstochowa, Poland

**Keywords:** Chemistry, Materials science, Optics and photonics, Physics

## Abstract

Detailed characterization of the lowest energy electronic excited states for dimethyl-acridine diphenyl-sulfone derivative (DMAC-DPS) in solid neat films has been performed by electroabsorption (EA) spectroscopy. The EA spectra were parametrized in the framework of Liptay theory, providing changes in the dipole moment and electronic polarizability. The extracted values of the corresponding parameters in the lowest energy optical absorption bands were assigned to molecular dipoles upon optical excitation associated with the intramolecular charge transfer (CT) from the electron-donating part (DMAC) to the electron-accepting part (DPS) of the molecule. The standard treatment of EA spectra based on Liptay theory was successfully reinterpreted applying the time-dependent density functional (TDDFT) theory, without additional assumptions regarding the shape of the EA signal, characteristic of the Liptay formalism. To correctly describe the EA spectrum, the TDDFT calculations have to be performed not for a single molecule, but for a dimer structure, established by the Monte Carlo (MC) calculations. Although the main EA features are ascribed to the excited states of intramolecular CT origin, the lowest energy excited states gain oscillator strengths due to the geometry distortion of the ground state or excited state interaction introduced by the dimer structure. The implications of these results for the first singlet excited state (S1) are discussed in the context of the thermally activated delayed fluorescence (TADF) emitters employed currently in organic light emitting diodes (OLEDs).

**Supplementary Information:**

The online version contains supplementary material available at 10.1038/s41598-025-29261-2.

## Introduction

**Fig. 1:**
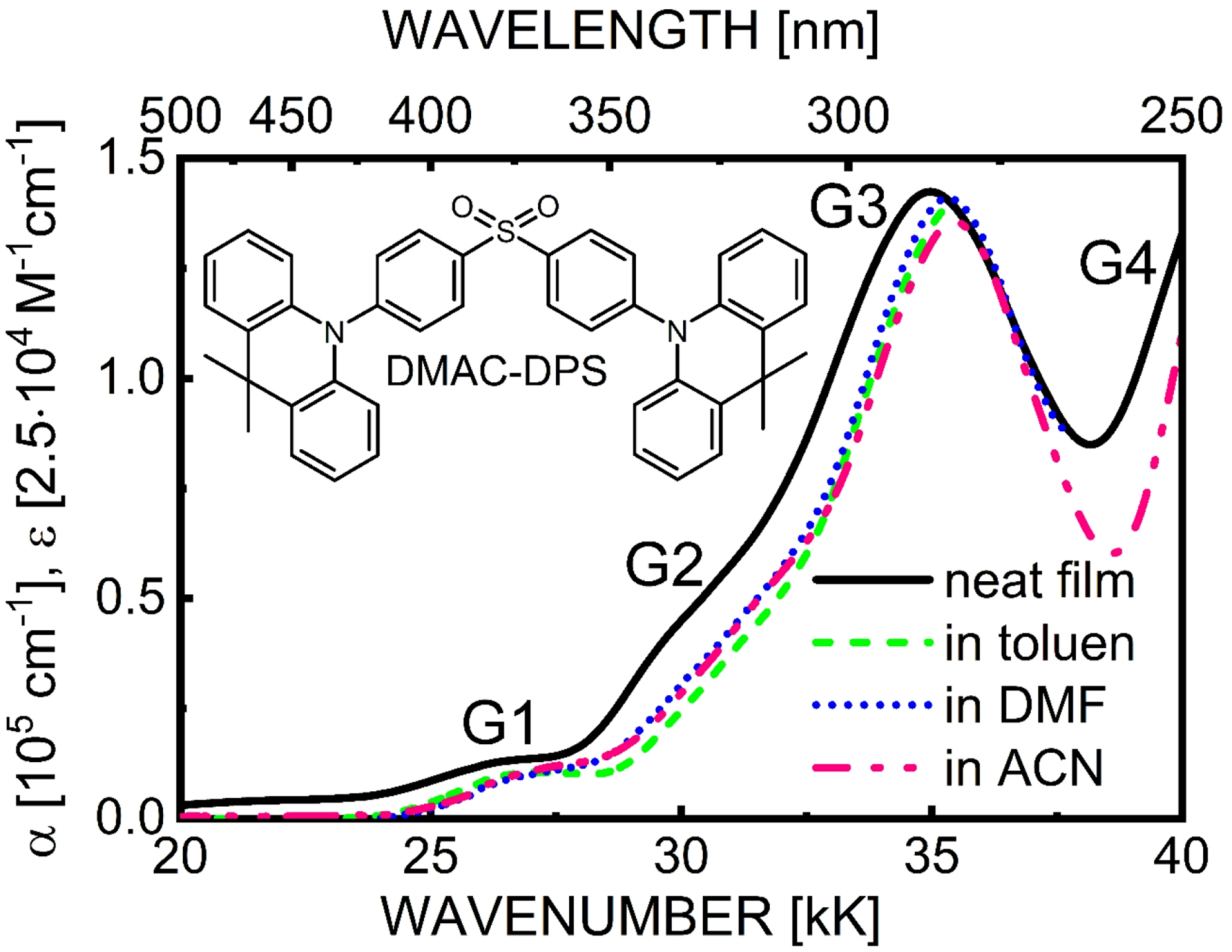
Optical density spectra of a solid film (solid line, thickness d = 74 nm) compared with spectra measured in dilute (10^− 5^ M) solvents (toluene - dashed line, DMF - dotted line, ACN - dash-dot line). Wavenumbers are given in kilokayser units (1kK = 1000 cm^− 1^). The inset shows the semi-structural formula of the DMAC-DPS molecule.

Bis[4-(9,9-dimethyl-9,10-dihydroacridine)phenyl]sulfone (DMAC-DPS, see inset of Fig. 1) has been widely studied as an efficient blue light emitter utilizing thermally activated delayed fluorescence (TADF) in organic light emitting diodes (OLEDs)^1–7^. TADF emitters have recently attracted great attention because they achieve 100% internal quantum efficiency (QE) of electroluminescence (EL) through harvesting triplet states via a reversed thermally activated intersystem crossing process (RISC) in organic fluorescent molecules, thus creating an efficient and cheaper alternative to so far applied phosphorescent EL emitters containing expensive iridium or platinum metals^8–10^. The activation energy for the RISC mechanism is determined by the energy gap (ΔE_ST_) between the lowest singlet (S1) and triplet state (T1). To minimize ΔE_ST_, various molecular design strategies are employed, but many of them rely on an intramolecular charge transfer (CT) mechanism between electron donor (D) and acceptor (A) units (DA-TADF mechanism), which is determined by the spatial separation of their frontier orbitals^1–5,11^. Two other TADF mechanisms have also been demonstrated: first one relying on the excited-state intramolecular proton transfer (ESIPT-TADF) in acridone based compounds with intramolecular hydrogen bonding^12^ and latter one hinging on the multi-resonance effect (MR-TADF) in fused planar polycyling aromatic compounds having electron-donating and electron-withdrawing atoms, which localizes frontier molecular orbitals on the atoms^13^. Although in the case of electrical excitation in OLEDs the emitting S1 state involved in the TADF mechanism is a relaxed LUMO excited state, its electronic characteristics can be generally associated with the Franck-Condon (“hot”) excited state formed instantaneously following the absorption of a photon by the molecule, however, the direct quantitative relationship between these singlet and triplet states involved in the RISC mechanism is not trivial^5^.

In the molecular system considered here, the degree of CT contribution to optical excited states can manifest as changes in the permanent dipole moment, which arises as a result of charge redistribution upon photoexcitation. An effective tool, specially dedicated to the study of such properties of molecules, is undoubtedly electroabsorption spectroscopy (EA), based on the Stark effect, in which the modifications in optical absorption are measured under the influence of a perturbation of an applied external electric field^14^. In fact, parametric analysis of EA spectra using the standard Liptay formalism^15^ allows for the assessment of electro-optical properties of excited states, such as changes after photoexcitation in the permanent dipole moment, electronic polarizability, and oscillator strength. As a side note, it is worth to mention that a charge redistribution can, in principle, be due to a proton charge displacement along the hydrogen bonds in ESIPT-TADF emitters, however this process being beyond the absorption time scale would not affect the EA spectra^16^.

In this work, combining EA measurements and quantum chemical calculations of the electronic structure, we have conducted a thorough and systematic analysis of the electronic excited states of the DMAC-DPS molecule in solid neat films. DMAC-DPS crystalline nanoaggregates have been recently implemented in efficient blue and white OLEDs^6,7^. Our goal is to determine the CT character of relevant excited states and to decide whether the CT attributes of the EA spectra of DMAC-DPS layers originate from a single molecule (intramolecular CT) or rather from molecular aggregates (intermolecular CT). Beyond to the conventional analysis of EA spectra within the framework of the Liptay theory^15^, we successfully report herein EA spectra of DMAC-DPS performed by quantum chemical calculations based on the time-dependent density functional method (TDDFT).

## Electroabsorption

Modification of an absorption (ABS) spectrum under action of an electric field is usually called as electroabsorption. The electric field $\overrightarrow{F}$ alters the energy E of non-degenerate electronic transition for a molecular dipole ensemble with a fixed spatial orientation, producing a shift Δ*E*($\:\overrightarrow{F}$), through the Stark effect:

1 $$\:\Delta{E}\left(\overrightarrow{F}\right)=\:-\overrightarrow{\varDelta\:{\upmu\:}}\cdot\overrightarrow{F}-\:\frac{1}{2}\overrightarrow{F}\circ\:\varDelta\:\boldsymbol{p}\circ\overrightarrow{F},$$

where $\:\overrightarrow{\Delta{\upmu\:}}$ is the change in permanent dipole moment, and **Δ***p* is the change in electronic polarizability tensor following photoexcitation. The Stark shift, Δ*E*($\overrightarrow{F}$), is assumed to be smaller than inhomogeneous bandwidth of the absorption band corresponding to a single well-separated vertical electronic transition. When the change in optical density (ΔD) caused due to the application of an electric field is not too large, which is usually the case, and assuming electric field independent oscillator strength of the electronic transition, then the quantity of Δ*D* (*E*, *F*) for an isotropic system can be determined from the averaged shift < Δ*E* > and broadening < (Δ*E*)^2^> of the absorption band and expressed by the following formula^17–19^:

2 $$\:{\Delta}D=\:\frac{1}{2}\left({\Delta}p\:\frac{\mathrm{d}D}{\mathrm{d}E}\:+\:{\upkappa\:}{\left({\Delta}{\upmu}\right)}^{2}\frac{{\mathrm{d}}^{2}D}{\mathrm{d}{E}^{2}}\right)\cdot\:{F}^{2}=B\left(E\right)\cdot\:{F}^{2}\:,$$

where the precise value of κ factor is governed by the specific details of averaging procedure applied to the molecular system and the electronic excited state features (non-degenerate or degenerate). Here, the *B*(E) function describes the photon energy dependence of optical density Δ*D*. The formula (2), which is basically consistent with the Liptay formalism^15^ for an unpolarized light beam traversing perpendicularly through a molecular layer with an external electric field generated by means of sandwich electrodes. In this article, for the evaluation of Δµ the value of κ = 1/3^17–19^ is assumed. The formula (2) assumes the electric field independent oscillator strength, which is characteristic for well-separated excited states^14^. According to this formula, the changes in the average (isotropic) electronic polarizability (${\Delta}p=\overline{{\Delta}p}$) and permanent dipole moment (${\Delta}\mu=\left|\overrightarrow{{\Delta}{\upmu\:}}\right|$) can be evaluated by juxtaposition of the measured Δ*D* spectra with the spectra of the first (D1) and second-order (D2) energy derivatives of optical density D, respectively. For example, in our group this way has been successfully characterized several materials commonly implemented in electronic devices, such as the organic complexes of aluminium(III) (Alq_3_)^20^, iridium(III) (Ir(ppy)_3_)^21^, platinum(II) (PtOEP)^22^, and ruthenium(II) (RuLp)^18,23^ to name just a few of the more well-known ones.

It is worth noting, however, that such an EA spectra analysis based on Liptay theory may not be generally sufficient to correctly describe all electronic transitions involved in the ABS spectrum. Firstly, degenerate (arising from molecular symmetry) or quasi-degenerate state must be treated with particular care. Moreover, in the case of many excited states, weakly resolved in ABS spectrum, the EA response is a multiparametric function, and its decomposition into individual components is not unambiguous, even if we neglect possible mixing of closely located excited states induced by the electric field. If this is the case, theoretical EA spectra should preferably be calculated directly from their definition as the difference between ABS spectra in and outside the electric field as demonstrated pioneerly for polyacene films by Petelenz group^24^ and more recently adopted by our group to ruthenium complexes^18,23^. Consequently, in the present paper, the results of EA spectra analysis based on Liptay formalism, were compared with the results obtained from the quantum chemical method TDDFT, free of Liptay theory restrictions.

## Results and discussion

Spectra of optical density (ABS) of DMAC-DPS solid films in the range from 20 kK to 40 kK (1kK = 1000 cm^− 1^) differ little from the quasi-identical spectra in dilute solution, independent of solvent (toluen, DMF, ACN) polarity (Fig. 1). It is clear that this ABS spectrum exhibits the typical for molecular solids, a bathochromic matrix shift of about 0.5 kK (0.06 eV), coming from a rather weak interaction between an excited molecule and its solid surroundings in the ground state. Moreover, intermolecular CT states, induced by electron transfer between adjacent molecules in the solid state, if they exist at all, their observable effect on the ABS spectrum is not recognized. In the ABS spectrum four bands can be distinguished, designated as G1, G2, G3 and G4 (compare also Fig. 5a). As confirmed by the TDDFT calculations presented below, the G1 and G2 bands, centered at approximately 26 kK (385 nm) and 30 kK (333 nm), respectively, can be attributed mainly to electronic transitions of CT origin, involving intramolecular charge transfer from the electron-donating DMAC part to the electron-withdrawing DPS part of the same molecule.

In turn, in the leading absorption band, centered at 35 kK (286 nm), the dominant transitions are ascribed to electronic states located at DMAC units. The broad G0 band introduced specifically to account for the low-energy tail of the ABS spectrum has an unclear origin, likely heavily modified by light scattering; therefore, this region of weak absorption was ignored, which does not significantly affect the further analysis of the results.

**Fig. 2:**
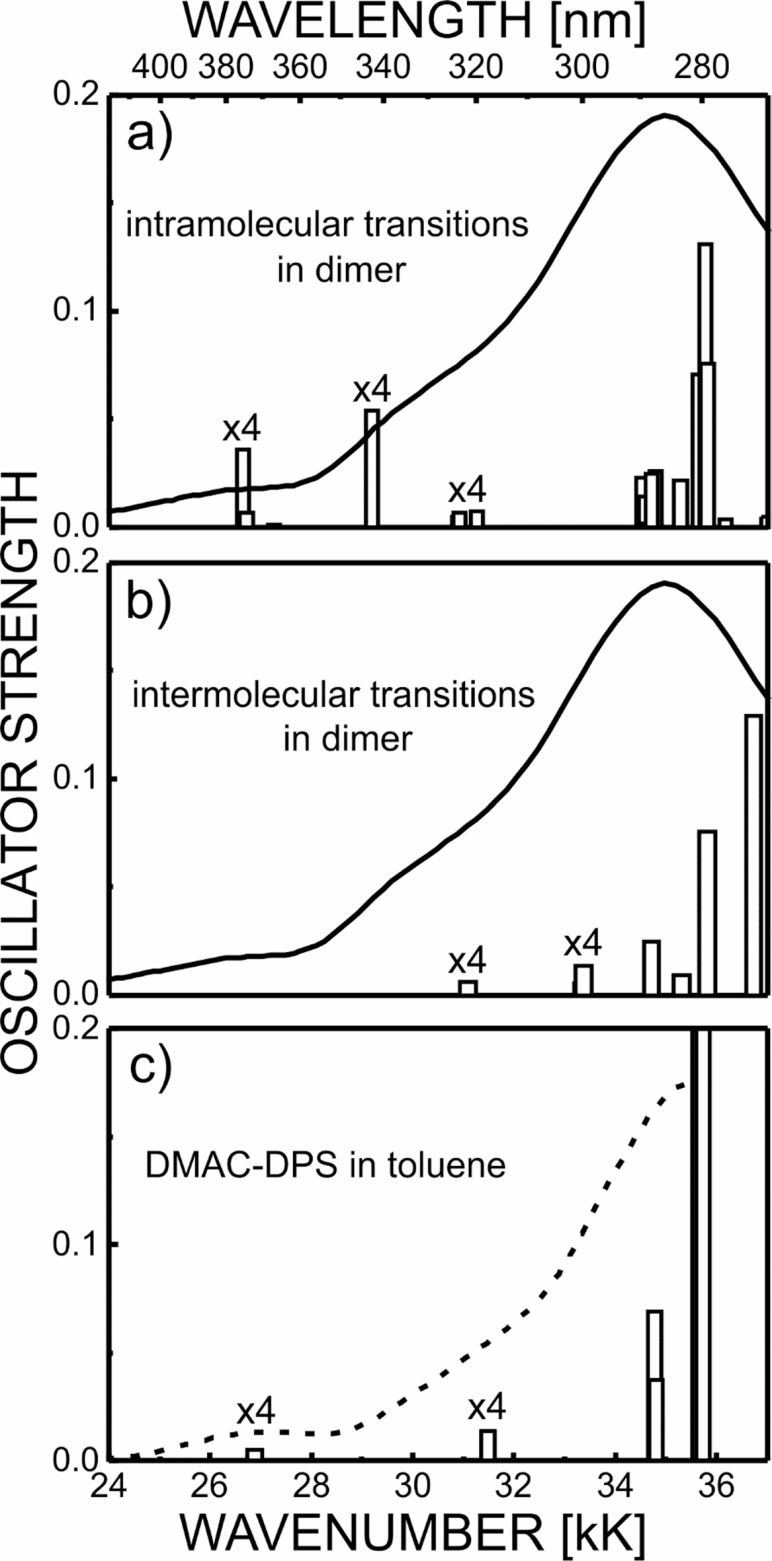
Experimental ABS spectra of a solid film (**a**,**b**) and 10^− 5^ M toluene solution (**c**) compared with the stick-bar spectra of oscillator strengths for intramolecular (**a**) and intermolecular (**b**) CT transitions for a dimer structure (**a**,**b**), and in a toluene solvent (**c**), calculated by the TDDFT method.

The origin of the G1 and G2 absorption bands requires further discussion. First and foremost, the TDDFT-calculated oscillator strengths for both, G1 and G2, single-molecule absorptions in vacuum are zero, and to obtain nonzero absorption intensity, the interaction of the molecule with its environment must be taken into account (Fig. 2). Second, as noted in this work, the excited states related to G1 and G2 absorption exhibit slightly different behavior. The G1 absorption associated with the lowest excited singlet state (S1) may arise due to distortion of the ground state geometry of the molecule, induced mainly by solid-state effects or, to a lesser extent, by interaction with solvent molecules. In the TDDFT method, the first effect is modelled by calculating the energy of excited states and their oscillator strength for the dimer structure determined by MC simulations (Fig. 2a,b). The second effect is shown in Fig. 2c, where the experimental ABS spectrum measured in a dilute toluene solution (10^− 5^ M) is juxtaposed with the stick-bar graph displaying the TDDFT oscillator strengths calculated for a single molecule interacting with a continuous solvent polarizable medium (see Section on TDDFT and MC calculations procedures for details). Moreover, according to TDDFT calculations performed for a single molecule in vacuum, but with the ground-state geometry taken from the structure of a dimer or a toluene solution, the oscillator strength for the G2 state in this case is still zero - in order to obtain a noticeable absorption in this spectral range, not only the ground state distortion must be taken into account, but also all the interactions of the excited states with the surrounding environment. In Fig. 2, oscillator strengths for intramolecular electronic transitions in the dimer (Fig. 2a) are compared with oscillator strengths for electronic transitions of intermolecular origin (Fig. 2b). It is clearly seen that absorption in the G1–G2 spectral range is dominated by intramolecular CT transitions. The electronic state pattern in the G3 spectral range, where multiple excited states of different origins (electron donor-located states, intramolecular and intermolecular CTs) coexist, is more complicated and more difficult to analyze reliably.

**Fig. 3:**
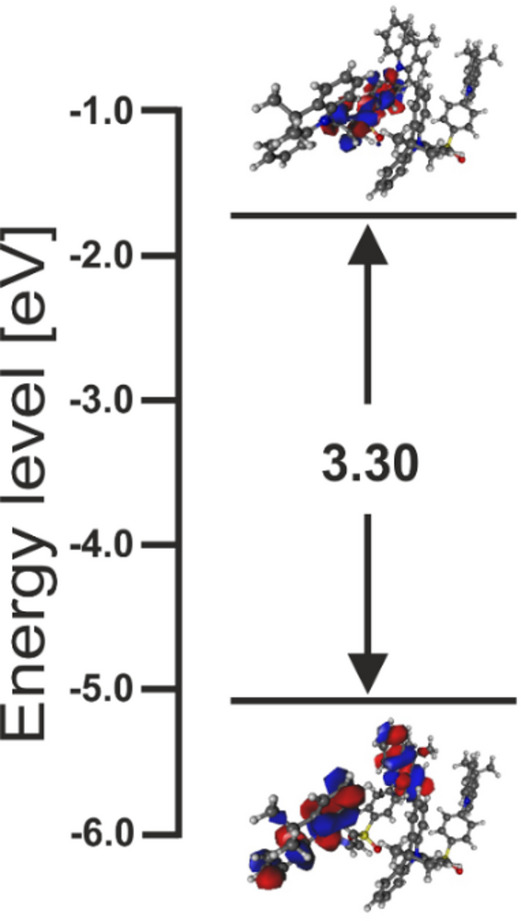
Schematic drawing of natural transitions orbitals (NTOs) for S0 and S1, and their energies for a dimer structure, with an optical energy gap of 3.30 eV associated with the lowest energy absorption band. For the molecular orbitals presented here, positive sign is in blue, negative in red.

For the operation mechanism of TADF emitters in efficient OLEDs, the characterization of the S1 state is important. Figure 3 shows a schematic drawing of natural transitions orbitals (NTOs) for S0 and S1, and their energies for a dimer structure, with an optical energy gap of 3.30 eV responsible for the G1 absorption, where the dimer configuration was chosen in the TDDFT calculations according to the ABS spectrum analysis above.

Additional information on excited states will be provided by electroabsorption spectroscopy, which allows the detection of weak electronic excitations that are usually hidden in an ordinary, rather broad ABS spectrum.

**Fig. 4:**
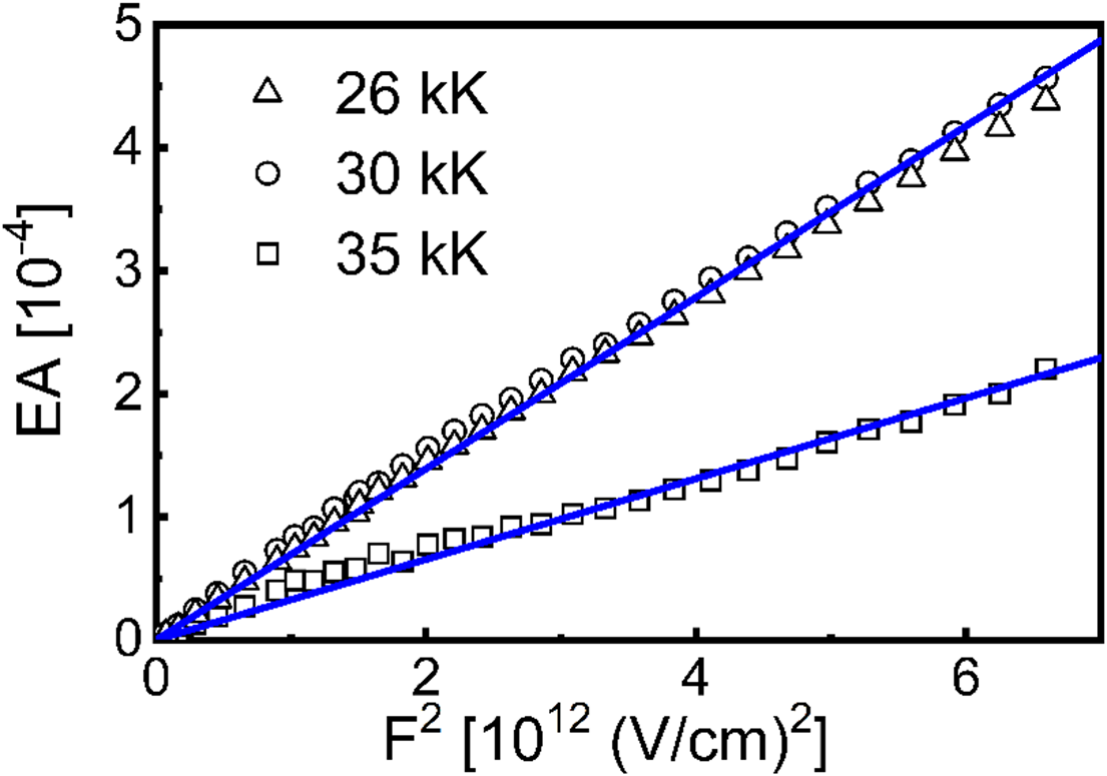
The EA signal vs. the second power of rms value of applied electric field (d = 74 nm) at various wavenumbers.

**Fig. 5:**
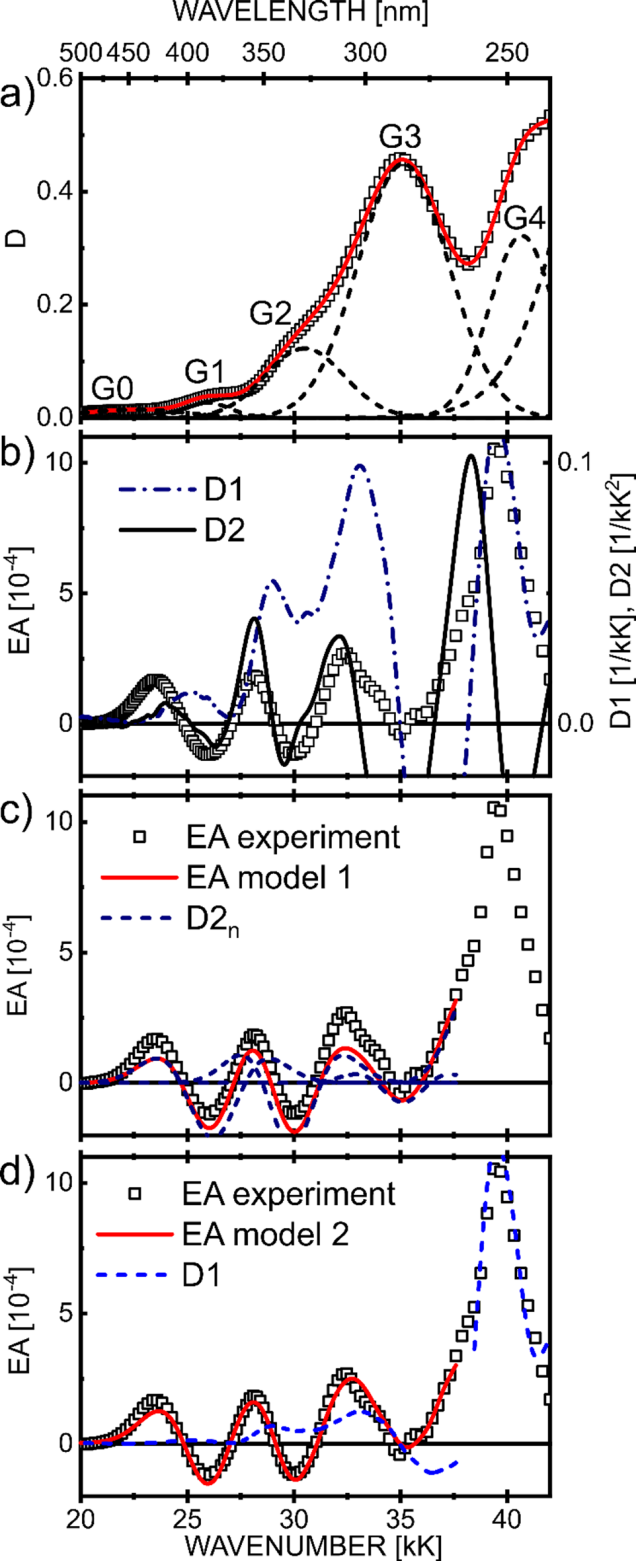
The experimental EA spectrum (squares, F_rms_ = 1.2 MV/cm) in comparison to the 1st (D1) and 2nd (D2) order energy derivatives of the global optical density (**b**), and the best fit-curve (solid line) obtained using model 1 (**c**) and model 2 (**d**). In part (**a**) of the figure the ABS spectrum (D, squares) was decomposed to a linear combination ($\:D=\sum_{n}{D}_{n}$, solid line) of the Gaussian components (dashed lines). In parts, (**c**) and (**d**), the contributions to the EA signal from the 2nd order (d^2^D_n_/dE^2^), individual Gaussian absorption derivative, and from the first order experimental absorption derivative (D1), respectively.

The EA signals are proportional to the second power of the applied electric field intensity (Fig. 4), confirming the Stark effect nature of electroabsorption in molecular solids. A representative example of the EA spectrum for a 74 nm-thick solid film in an electric field with the root mean square (rms) value of electric field intensity, F_rms_= 1.2 MV/cm, is shown in Fig. 5b–d using squares. In the G1–G3 spectral range the shape of EA spectrum roughly mimics the absorption 2nd order derivative (Fig. 5b, D2), showing three distinct negative lobe minima at around 26 kK, 30 kK and 35 kK, which can be ascribed to the Δµ term in formula (2) due to the nonzero change in the permanent dipole moment after the vertical transition from the ground state to the excited state. On the contrary, the EA signal, in the spectral range above 37 kK involving the G4 band, is better reproduced by the absorption first derivative (Fig. 5b, D1). The following quantitative analysis applying Liptay and quantum chemical methods supports the idea that the EA spectrum of a solid film is mainly due to intramolecular charge redistribution with less important contributions from intermolecular CT transitions.

Next, a conventional Liptay analysis of EA spectra was step by step carried out. The ABS spectrum was resolved ($\:D=\sum_{n}{D}_{n}$) into Gaussian components (Fig. 5a), and then fitting the EA spectrum using Eq. (6) in model 1, including only permanent dipole moments of excited states, or Eq. (7) in model 2, which additionally takes into account arbitrarily the same change in polarizability for the considered spectral region (see discussion below). The best fitting gained for model 1 is shown in Fig. 5c with solid line, where the D2_*n*_ (D2_*n*_ = d^2^D_*n*_/dE^2^) contributions (*n* = 1,2,3) to the EA signal are also shown with dashed lines. Further, in Fig. 5d, the best EA results obtained from model 2 (solid line) are compared with the first derivative term (a⋅dD/dE, dashed line) in Eq. (7). The values of molecular parameters for excited states grouped in G1-G4 bands are collected in Table 1 (see also Table 1S for more details), whereby the given values take into account the local electric field correction *f* factor, it means they refer actually to *f*·Δµ and *f*^2^⋅Δ*p*. The values in the table have been rounded to two significant digits, i.e. with a relative error of about 10%. The excited-minus-ground permanent dipole moments, Δµ_*n*_, were calculated taking the maximal intensities *D*_0*n*_ of the Gaussian bands depicted in Fig. 5a. First, we can see that the dipole moment change, Δµ_3_, for the G3 band, associated with the excited states well confined to rather small volume of DMAC units, are at least several times smaller than the Δµ_1_ and Δµ_2_ dipole moments related to the G1 and G2 spectral ranges in which intramolecular CT transitions involving electron transfer from the electron donor DMAC to the electron acceptor DPS unit take part. We also note here that a dipole moment of 13 debyes indicates the complete electron transfer at a distance of 2.6 Å, while the distance between the centers of DMAC and DPS parts of the molecule is estimated to be 7.5 Å, indicating the moderate coupling of the transferring orbitals. Second, the TDDFT average value of Δµ for three transitions occurring in the spectral area of the G1 band (see Fig. 2a) is about 15 debyes. The changes in dipole moment and polarizability, calculated applying Liptay theory and TDDFT methods, were estimated for the G1–G4 bands and juxtaposed in Table 1. For the G1 band, the molecular parameters, Δµ and Δ*p*, are in good agreement, and for the G4 band, the change in polarizability Δ*p* also shows suprising agreement with almost neglected Δµ terms. For the remaining bands (G2 and G3), the correlations between the parameters are not as good.

The value of *f*^2^·Δ*p* received from model 2 in the G1–G3 spectral range is about 20 Å^3^, several times less than the ground state electronic polarizability estimated to be 160 Å^3^ by the TDDFT method. For comparison, the value of this parameter in the spectral range of the lowest excited states for anthracene dissolved in the polymer matrix^25^ is 36 Å^3^, and for solid films of tris(2-phenylpyridine) Ir(III) complex^21^, Ir(ppy)_3_ − 20 Å^3^, or a Pt(II) octaethylporphyrin^22^, PtOEP − 25 Å^3^, all of which were estimated.

based on EA measurements using the Liptay formalism. In the spectral area of the G4 band, above 37 kK, where the 2nd derivative of the ABS spectrum can be practically neglected, the polarizability change is much larger, amounting to 150 Å^3^. The situation is more complicated when considering the isotropic TDDFT values of Δp (the average of the diagonal elements of the polarizability tensor), which are relevant for solid films. For excited states that are relatively well isolated on the energy scale, the values of this parameter are variable and amount to several tens Å^3^. However, the quasi-degenerate states (for example, pairs of electronic states: S1 and S2, S5 and S6, S10 and S11 or S17 and S18) constitute a separate case, where the polarizability values reach as much as 15 thousand Å^3^, with the polarizability of the higher energy state always being negative. This is completely understandable, because in the two-state model of the electronic system, the higher-energy state is characterized by negative polarizability, the absolute value of which is greater the smaller the energy gap between the states. However, in broadband EA spectroscopy, such rapidly changing components are difficult to detect within a more moderate signal. In addition, it is well documented in literature that for EA spectrum of polar molecules, dominated by the absorption 2nd order derivative terms, the reliable determination of electronic polarizability is usually difficult to perform. For example, the authors of the paper^26^ observed EA spectra with relatively small and variable first derivative terms for anthracene-9-carboxylic acid (ACA) molecules attached to TiO_2_, which made it impossible to determine the change in electronic polarizability at all. Another issue arises when the solid DMAC-DPS layers are not fully isotropic, in which case the tensor nature of the electronic polarizability must also be taken into account, as confirmed by TDDFT calculations for a single molecule.

**Table 1:** The values of dipole moment changes, *f·*Δµ (in debyes), polarizability changes, *f*^2^·Δ*p* (in Å^3^) in the spectral area of G1–G4 bands as determined according to Liptay formalism (using model 1 and model 2) and TDDFT method. The uncertainty of parameters is around 10%. The $\:\:{\Delta}{\mu}_{\mathrm{a}\mathrm{v}}$ and $\:{\Delta}{p}_{\mathrm{a}\mathrm{v}}$ designate the arithmetic mean of $\:{\Delta}{\mu}_{n}$ and $\:{\Delta}{p}_{n}^{\mathrm{i}\mathrm{s}\mathrm{o}}$ calculated for the *n*th band (see Table 2S), respectively.

Band	Model 1	Model 2		TDDFT	
	*f*·Δµ	*f·*Δµ	*f*^2^⋅Δp	Δµ_av_	Δp_av_
G1	13	14	20	15	18
G2	6.0	8.0	20	13	290
G3	1.6	4.4	20	12	57
G4	–	–	150	8	144

**Fig. 6:**
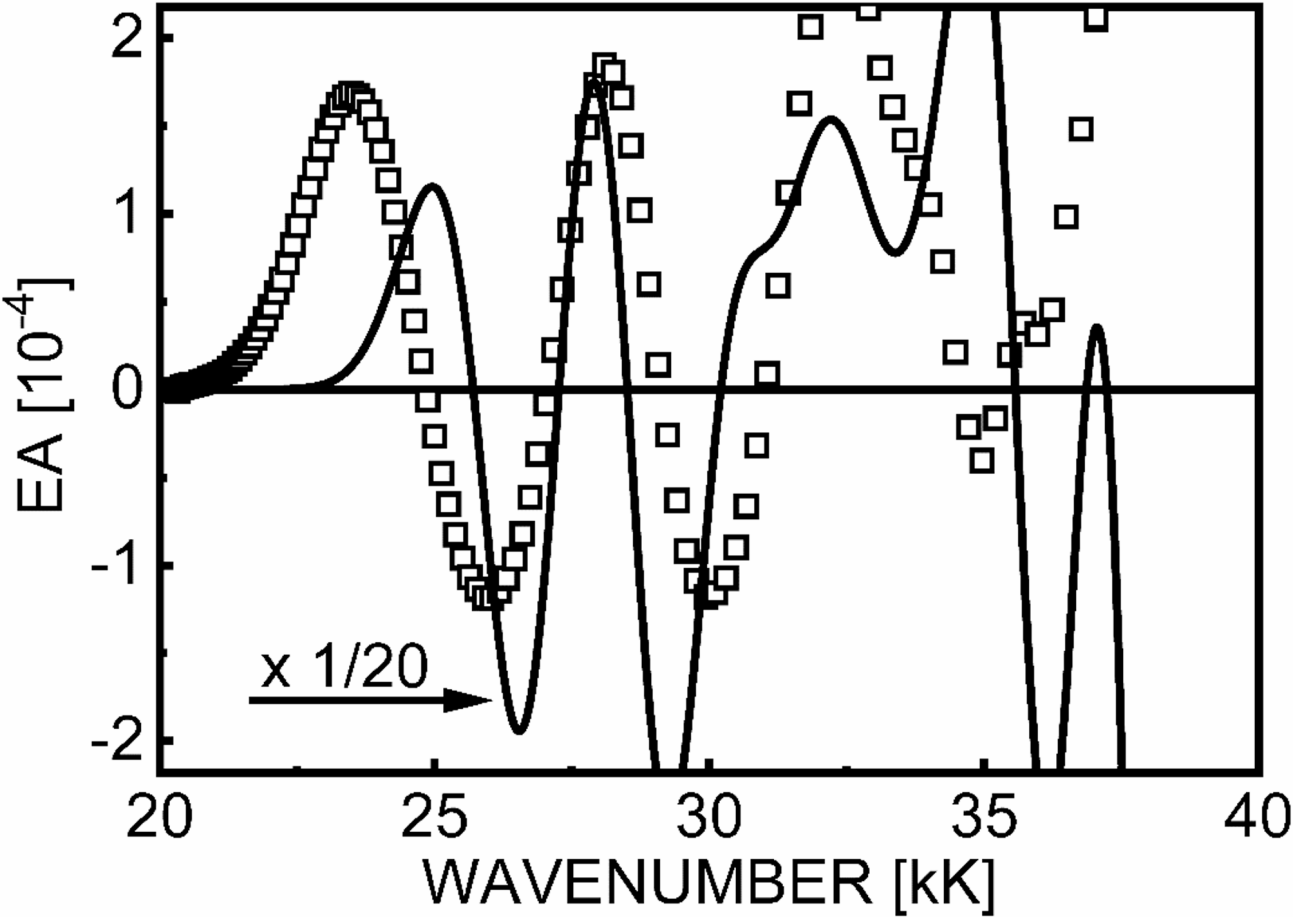
A comparison of theory (TDDFT) with experiment. The EA signals were calculated at F = 5.14 MV/cm (solid line, *w* = 2.0 kK) and the experimental EA data (squares) measured at F_rms_ = 1.2 MV/cm are taken from Fig. 5b.

The standard decomposition of the EA spectrum into a multicomponent linear combination of energy derivatives of individual ABS profiles, based on Liptay formalism, is difficult to perform reliably in a situation where many excited states, especially those located closely, coexist on the energy scale. Therefore, the above estimation of the Δµ and Δ*p* parameters should not be considered too restrictive. A much better and more consistent way to unravel the interaction patterns of electronic states is to reproduce the EA spectrum applying the TDDFT, as demonstrated earlier for organic Ru complexes^18,23^. As can be seen in Fig. 6, the experimental EA spectrum in the considered spectral range is more or less described by TDDFT results (vide infra, TDDFT Section). The values of theoretical EA signals calculated at 5.14 MV/cm are around 20 times higher than the experimental EA signals measured for 1.2 MV/cm. Given the quadratic dependence of EA signals on the electric field strength (see Fig. 4), and scaling the calculated EA signals to the experimental electric field of 1.2 MV/cm, very good agreement theory with experiment has been received. The assumed width of Gaussian bands, *w* = 2 kK = 2000 cm^− 1^, is somewhat smaller than those obtained from the experimental global ABS spectrum (Fig. 5a), but has a reasonable value given, for example, the vibrational aromatic ring stretching modes of 1300–1500 cm^− 1^ in organic compounds. Therefore, the relatively good reproduction of both the spectral shape and the magnitude of the EA signal represents a significant achievement, taking into account a number of approximations used in the calculations, in particular assumption that the Lambert–Beer law can extended to the solid state.

## Conclusions

The involvement of charge transfer in the lowest singlet excited state S1 is an important issue in properly explaining the operation of DA-TADF emitters in OLEDs. As mentioned in the introduction, under electrical excitation in OLEDs, the relaxed S1 state is responsible for light emission, but its main CT properties can already be observed essentially in the absorption of photons by the Franck-Condon S1 excited state, although the relationship between these states requires careful analysis^5^. Accordingly, the lowest excited states of DMAC-DPS were comprehensively analyzed by EA spectroscopy. The conventional analysis of EA spectra performed on the grounds of Liptay theory was successfully compared with the EA spectra obtained applying the TDDFT theory, without referring to the energy derivative shape of the EA signal from individual electron transitions, characteristic of the Liptay treatment of electroabsorption. The values of the changes in dipole moment Δµ upon photoexcitation, extracted from the EA spectrum, were assigned to the intramolecular charge transfer from the DMAC to the DPS part of the molecule. In particular, the value of Δµ for the S1 state was estimated on 13–15 debyes, indicating the moderate coupling of the transferring DMAC and DPS orbitals. Although the main features observed in the EA spectrum can be attributed to intramolecular CT states, the first eight singlet excited states of the lowest energy (S1-S8) gain oscillator strengths due to the distortion of the ground state geometry or the interaction of the excited state introduced by the surrounding environment. This sensitive environmental influence on the S1 state should also be carefully considered when developing the emission mechanism of OLEDs with TADF emitters. Satisfactory reproduction of the EA spectrum was achieved by performing TDDFT calculations for the dimer structure, which suggests that the main features of the excited states were captured. However, in the low-energy spectral region (below 35 kK) intermolecular charge transfer process is not efficient enough to observe the relevant CT states in the EA spectrum. On the other hand, in the higher energy region (above 35 kK), due to the involvement of many excited states of intra- and intermolecular origin, the EA mechanism is too complicated to give a reliable interpretation.

## Methods

### Sample details

The DMAC-DPS (CAS number 1477512-32-5) was purchased from Angene Int. Ltd. Optical absorption was recorded on quartz glass substrates. In EA measurements, samples with a sandwich configuration, quartz/Al/organic layer/Al, with aluminum electrodes, were used. The solid neat films of DMAC-DPS were formed by thermal deposition in vacuum ($\:{10}^{-3}$ Pa) at an average rate of 1 nm/s. Thickness of layers were measured by a profiler (Tencor, model Alpha Step 500).

### EA measurements

For the basics of the modulation technique used and the details of the experimental setup, the reader is referred to the more comprehensive relevant literature^23,27–31^. The EA signals presented in the graphs are defined as:

3 $$\:EA\equiv\:\frac{{I}_{2\omega\:}}{{I}_{0\omega\:}},$$

where $\:{I}_{2\omega\:}$ is the rms value of the 2nd harmonic-Fourier component measured by a lock-in amplifier, and $\:{I}_{0\omega\:}$ is the value of the zero-order Fourier component measured by an electrometer, for the beam light transmitting through the sample, detecting with a photomultiplier (EMI 9781).

The relationship between the EA signals (3) and the frequently used quantity ΔD (2) by others is as follows:

4 $$\:EA=\frac{\mathrm{l}\mathrm{n}\left(10\right)}{2\sqrt{2}}\:{{F}_{e}}^{2}\cdot\:B\left(E\right)\:=\:\:\frac{\mathrm{ln}\left(10\right)}{2\sqrt{2}}{\Delta}D\:,$$

where *F*_e_ = *f*⋅*U*/*d*, and *U* is the voltage amplitude applied to the sample of thickness *d*. The local electric field correction factor *f*, in the Lorentz spherical cavity approximation, *f* = (ε + 2)/3, which for a typical dielectric constant, ε = 3–4, leads to a field enhancement factor, *f* = 1.7-2.0. In the simplest approximation, neglecting the effects of solid state polarization, one should assume *f* = 1.

### Liptay formalism for EA spectra

Details of used numerical procedures can be found in reference^23^. The decomposition of ABS spectra ($\:D=\sum_{n}{D}_{n}$) was performed using Gaussian functions:

5 $$\:{D}_{n}={D}_{0n}\:\mathrm{e}\mathrm{x}\mathrm{p}\left[-4\:\mathrm{l}\mathrm{n}2\:{\left(E-{E}_{n}\right)}^{2\:}/\:{{w}_{n}}^{2}\:\right]\:,$$

where *w*_*n*_ = FWHM (full width at half maximum), *E*_*n*_ is the energy, and *D*_0*n*_ – the maximum optical density of the *n-*th electronic transition.

The EA spectra were analyzed within the Liptay formalism by fitting the experimental data to theoretical curves derived from Eq. (6) for model 1, and from Eq. (7) for model 2:

6 $$\:EA\:=\:\sum_{n}{b}_{n}\:\frac{{d}^{2}{D}_{n}}{\mathrm{d}{E}^{2}}\:,$$

7 $$\:EA\:=\:a\:\frac{\mathrm{d}D}{\mathrm{d}E}\:+\:\sum_{n}{b}_{n}\:\frac{{\mathrm{d}}^{2}{D}_{n}}{\mathrm{d}{E}^{2}}\:.$$

Model 1 accounts solely for the second-derivative contributions weighted by the *b*_*n*_ factors depending on dipole moment differences (Δµₙ). Alternatively, model 2 includes an additional first-derivative term of the total ABS spectrum (dD/dE), scaled by a single factor (*a*) defined by the same difference in electronic polarizability (Δ*p*) for all excited states involved in the considered spectral range. In the fitting procedure following parameters, *a* and *b*_*n*_, energy positions (*E*_*n*_) and bandwidths (*w*_*n*_) were optimized. From the extracted values of *a* and *b*_*n*_ coefficients, taking the amplitudes D_0*n*_ from the ABS spectrum (Fig. 5a), the Δ*p* and Δµ_*n*_ molecular parameters were calculated (Table 1). Due to the common practice in the literature of providing dipole moments and electronic polarizability in the older system of units (respectively, in debyes and Å^3^), for the reader’s convenience, conversion factors to the SI unit system are given: 1D = 3.34 · 10^–30^ C m, and 1Å^3^ = 1.11 · 10^–40^ F m^2^.

In all spectral graphs, the wavenumber is plotted on the abscissa axis and expressed in kilokaysers units (1 kK = 10^3^ cm^−1^).

### Monte Carlo simulations

To investigate the environmental effects and mutual interactions between DMAC-DPS molecules a molecular model was constructed based on a cubic unit cell containing two DMAC-DPS molecules. The unit cell edge was set to 11.8 Å, corresponding to a theoretical density of 1.22 g/cm^3^, consistent with the estimated bulk density of the compound.

The simulations were performed under periodic boundary conditions to mimic the behavior of the bulk phase and eliminate surface effects. The five initial structures were built and subsequently optimized using the Forcite module within the Materials Studio software package. The structures were constructed with different arrangements of DMAC-DPS molecules to avoid the error of constructing a specific atomic configuration. Geometry optimization was carried out employing the Universal Force Field (UFF)^32^. The convergence criteria were set to 10^−4^ kcal/mol for total energy and 10^−3^ kcal/mol Å for the atomic forces. From the obtained structures, the one with the lowest total energy was selected.

To achieve thermodynamic equilibrium, Monte Carlo simulations were performed, allowing for adequate sampling of configurational space and relaxation of the system under equilibrium conditions. For electrostatic interactions, Ewald summation with 10^–6^ kcal/mol precision was used. The cutoff radius was set to 11 Å according to the length of the unit cell, and periodic boundary conditions were applied in all dimensions. Simulations were performed with 5⋅10^3^ cycles for initialization and 10^5^ cycles for production runs. The obtained structure was used to calculate the electron and optical properties of the DMAC-DPS dimer-like structure. Before proceeding with the essential quantum chemical calculations, the dimer was reoptimized using the same methods as it was applied for the separated DMAC-DPS molecule.

### TDDFT calculations

The geometry of the DMAC-DPS molecule was optimized by minimizing its total energy applying density functional theory (DFT) with the MN15 functional^33^ and the def2-TZVP basis set^34^ (DFT/MN15/def2-TZVP). It should be noted that the MN15 functional, although not range-separated and therefore not containing the full 100% asymptotic exchange, was selected based on its balanced performance for a broad-spectrum range. While range-separated hybrid functionals (e.g., CAM-B3LYP, ωB97X-D, LC-ωPBE)^35–37^ are generally recommended for systems dominated by long-range CT excitations, previous benchmark studies have shown that MN15 performs reliably for local and short-range CT excitations due to its well-balanced treatment of exchange and correlation effects^33,38^. Geometry optimizations were performed both in vacuum and in toluene. In the latter case, solvent effects were modeled using the polarizable continuum model (PCM)^39^. The SCF convergence was not higher than 10^− 8^ Hartree, but the geometry convergence was reached with the parameters RMS force ≤ 10^− 4^ Hartree/Bohr and the RMS displacement ≤ 10^− 3^ Å. All DFT calculations were carried out using the Gaussian 16 software package^40^.

Considering the lack of heavy atoms in the structure of DMAC-DPS, the calculations were carried out exclusively for singlet states. Transition energies were computed using the iterative Davidson algorithm^41^ with a convergence threshold of 10^−12^ Hartree. The vertical excitation energies and oscillator strengths for the lowest 40 singlet excited states were computed applying time-dependent DFT (TDDFT) at the DFT/MN15/def2-TZVP level. Calculations were performed for DMAC-DPS monomers in vacuum and in toluene, as well as for the DMAC-DPS dimer in vacuum. All TDDFT calculations were based on geometries optimized in the ground state.

The EA spectra in TDDFT method were obtained as the difference of the ABS spectra calculated in the presence and without of an electric field. To overcome numerical noise problems, TDDFT calculations were performed at an electric field of 0.001 au = 5.14 MV/cm, i.e., about 4.3 times larger than the experimental electric field of magnitude 1.2 MV/cm. The external field was applied along each of the six Cartesian directions (− x, x, −y, y, −z, z), where the *z* axis is directed along the ground state dipole moment of the molecule. The resulting spectra were averaged to account for the isotropic orientations of molecules in the sample. The ABS spectra were formed as a sum ($\:D=\sum_{n}{D}_{n}$) of *n* Gaussian bands, *D*_*n*_ (formula 5), where *E*_*n*_ is the calculated energy, and the amplitude *D*_*0n*_ is determined from the calculated oscillator strength *f*_*n*_, according to the assumed Lambert–Beer law from the liquid solution up to the solid state^18,42^:

8 $$\:{D}_{0n}=c\:l\:\frac{{f}_{n}}{4.6\cdot{10}^{-9}{\:w}_{n}}\:,$$

where *c* is the molar concentration (in mol/dm^3^), *l* is the thickness (in cm) of the layer, and *w*_*n*_ is the bandwidth (in cm^− 1^). In this work, the thickness *l* = 74 nm and the width, *w*_*n*_*= w* ≅ 2.0 kK, was arbitrarily assumed for all contributing bands, which roughly correspond to the Gaussian bands displayed in Fig. 5a.

By taking an experimental molar extinction coefficient, ε = 3.2 · 10^4^ M^− 1^cm^− 1^ for the liquid acetonitrile solution (ACN), and the linear absorption coefficient, α = 1.4 · 10^5^ cm^− 1^ for the solid layer at the main absorption maximum (λ = 284 nm), the molar concentration, *c* = 1.9 mol/dm^3^ was obtained. Unfortunately, this value is difficult to verify because the crystal structure of DMAC-DPS is currently unknown. It is worth mentioning, however, that a similar evaluation procedure to estimate *c* concentration from absorption data, has been successfully applied to ruthenium dyes^23^, where *c* values can be directly determined from the crystal structure. In comparison, for molecules of similar molar mass as DMAC-DPS (500–600 g/mol), the crystal structure values of 2.0 mol/dm^3^ and 2.3 mol/dm^3^ are significant, for bipyridine ruthenium(II) complex, Ru(bpy)_3_^43^ and N, N′-diphenyl-N, N′-bis(3-methylphenyl)-1,1′-biphenyl-4,4′-diamine, TPD^44^, respectively.

Additionally, the changes in permanent dipole moment (Δµ) and electronic polarizability (Δ*p*) after photoexcitation to the first singlet excited states were computed for the DMAC-DPS dimer at the DFT/MN15/def2-TZVP level (see Tables 1, and 2S). The ground-state geometry used in these calculations was taken from Monte Carlo simulations performed under periodic boundary conditions to model realistic molecular packing.

## Supplementary Information

Below is the link to the electronic supplementary material.

Supplementary Material 1

## Data Availability

All the data are available on request from the corresponding author.
