# Supplementary material for: Optical excited states of archetypical blue light emitter, dimethyl-acridine diphenyl-sulfone derivative (DMAC-DPS) in solid films, studied by electroabsorption spectroscopy
Source: Sci Rep. 2025 Nov 21;15:44963. doi: 10.1038/s41598-025-29261-2 (PMC12749668; doi:10.1038/s41598-025-29261-2)
Supplement: Supplementary file 1 — Supplementary Material 1 [file 41598_2025_29261_MOESM1_ESM.docx]

SUPPLEMENTARY MATERIALS

Optical excited states of archetypical blue light emitter, dimethyl-acridine diphenyl-sulfone derivative (DMAC-DPS) in solid films, studied by electroabsorption spectroscopy

Daniel Pelczarski^1*^, Malgorzata Makowska-Janusik^2^, Waldemar Stampor^1^

^1^ Department of Molecular Photophysics, Institute of Applied Physics and Mathematics, Gdańsk University of Technology, 11/12 Narutowicza str., 80-233 Gdańsk, Poland;

^2^ Faculty of Science and Technology, Jan Dlugosz University, Al. Armii Krajowej 13/15, 42-200 Czestochowa, Poland;

* corresponding author e-mail address: daniel.pelczarski@pg.edu.pl

**Table 1S.** Results of fitting model parameters for EA spectra of the DMAC-DPS molecule. The uncertainty of parameters Δμ and Δ*p* is around 10%. Spectral positions are expressed in kilokaysers (1kK = 1000 cm^‑1^).

| **Band** | **ABS** | **EA Model 1** | | **EA Model 2** | | |
| --- | --- | --- | --- | --- | --- | --- |
|  | **Posit.** | **Posit.** | ***f*·Δµ** | **Posit.** | ***f·*Δµ** | ***f*^2^·Δp** |
|  | **[kK]** | **[kK]** | **[debye]** | **[kK]** | **[debye]** | **[Å^3^]** |
| **G0** | 22.53 | - | - | - | - | - |
| **G1** | 25.94 | 26.20 | 13 | 26.14 | 14 | 20 |
| **G2** | 30.49 | 29.90 | 6.0 | 29.92 | 8.0 | 20 |
| **G3** | 35.12 | 35.11 | 1.6 | 35.19 | 4.4 | 20 |
| **G4** | 40.68 | - | - | - | - | 150 |

**Model 1:**

$$EA=\sum_{n} b_{n} \frac{d^{2}D_{n}}{dE^{2}},$$

**Model 2:**

$$EA = a \frac{dD}{dE} + \sum_{n} b_{n} \frac{d^{2}D_{n}}{dE^{2}} .$$

In model 1, the dominant contribution of second derivative components associated with the permanent dipole moments (Δμ*_n_*) was assumed. In model 2, the first derivative (d*D*/d*E*) of total (experimental) absorption spectrum (*D*) was added with a single scaling factor (*a*), assuming the same electronic polarizability change (Δ*p*) for all transitions considered. The fitting procedure was performed using a non‑linear least‑squares method based on the Levenberg-Marquardt algorithm.

**Table 2S.** The excitation energies for singlet states (in eV, nm, kK units), oscillator strengths (f), changes in dipole moment (Δµ) and electron polarizability (Δp^iso^) calculated by TDDFT method.

| **SYMBOL** | **E** | **λ** | $\tilde{\boldsymbol{\nu}}$ | **f** | **Δµ ^a)^** | **Δp^iso^ ^b)^** | **BAND** |
| --- | --- | --- | --- | --- | --- | --- | --- |
|  | **[eV]** | **[nm]** | **[kK]** |  | **[debye]** | **[Å^3^]** |  |
| **S0** | - | - | - | - | - | - |  |
| **S1** | 3,302 | 375,4 | 26,64 | 0,0090 | 14,63 | 1188 | G1 |
| **S2** | 3,311 | 374,4 | 26,71 | 0,0017 | 15,42 | -1151 |  |
| **S3** | 3,379 | 366,9 | 27,25 | 0,0002 | 15,59 | 17,51 |  |
| **S4** | 3,619 | 342,5 | 29,19 | 0,0134 | 15,18 | 35,04 | G2 |
| **S5** | 3,832 | 323,6 | 30,90 | 0,0012 | 12,48 | 5481 |  |
| **S6** | 3,832 | 323,5 | 30,91 | 0,0016 | 11,58 | -4138 |  |
| **S7** | 3,854 | 321,7 | 31,09 | 0,0015 | 17,35 | 40,55 |  |
| **S8** | 3,875 | 320,0 | 31,25 | 0,0018 | 10,89 | 37,03 |  |
| **S10** | 4,134 | 299,9 | 33,34 | 0,0014 | 25,96 | 1850 | G3 |
| **S11** | 4,138 | 299,6 | 33,37 | 0,0034 | 6,820 | -1628 |  |
| **S12** | 4,281 | 289,6 | 34,53 | 0,0230 | 14,34 | 3636 |  |
| **S13** | 4,289 | 289,1 | 34,59 | 0,0140 | 1,227 | 441,1 |  |
| **S14** | 4,293 | 288,8 | 34,63 | 0,0018 | 14,94 | -802,8 |  |
| **S15** | 4,304 | 288,0 | 34,72 | 0,0495 | 0,7050 | 126,3 |  |
| **S16** | 4,313 | 287,4 | 34,79 | 0,0258 | 14,71 | -2389 |  |
| **S17** | 4,374 | 283,4 | 35,28 | 0,0219 | 12,52 | 4989 |  |
| **S18** | 4,378 | 283,2 | 35,31 | 0,0090 | 40,41 | -4027 |  |
| **S19** | 4,418 | 280,6 | 35,63 | 0,0704 | 2,140 | -95,72 |  |
| **S20** | 4,435 | 279,6 | 35,77 | 0,1310 | 1,318 | 406,5 |  |
| **S21** | 4,438 | 279,4 | 35,80 | 0,0757 | 2,870 | 1205 |  |
| **S22** | 4,440 | 279,2 | 35,81 | 0,1517 | 2,306 | -3514 |  |
| **S23** | 4,485 | 276,4 | 36,17 | 0,0035 | 36,64 | 374,4 |  |
| **S24** | 4,554 | 272,2 | 36,73 | 0,1292 | 4,594 | 288,7 |  |
| **S32** | 4,769 | 260,0 | 38,46 | 0,0916 | 3,795 | 172,0 | G4 |
| **S33** | 4,785 | 259,1 | 38,60 | 0,0720 | 5,020 | -689,6 |  |
| **S36** | 4,837 | 256,3 | 39,01 | 0,0044 | 30,19 | -21,85 |  |
| **S37** | 4,843 | 256,0 | 39,06 | 0,0858 | 1,817 | -531,4 |  |
| **S40** | 4,954 | 250,3 | 39,96 | 0,1627 | 1,508 | 1791 |  |

1. $\Delta\mu_{n}=\sqrt{\left( \mu_{\mathrm{nx}}-\mu_{0x} \right)^{2}+\left( \mu_{\mathrm{ny}}-\mu_{0y} \right)^{2}+\left( \mu_{\mathrm{nz}}-\mu_{0z} \right)^{2}}$ ;
2. $\Delta p_{n}^{\mathrm{iso}}=\frac{\left( p_{\mathrm{nx}}-p_{0x} \right)+\left( p_{\mathrm{ny}}-p_{0y} \right)+\left( p_{\mathrm{nz}}-p_{0z} \right)}{3}$ ;

The X, Y, Z coordinates of the DMAC-DPS molecule optimized in vacuum applying the DFT/MN15 method. The coordinates X, Y, Z are given in angstroms.

C -1.384242 2.537951 -0.000080

C -1.909526 2.113033 -1.215063

C -1.908708 2.111785 1.214832

C -2.989285 1.242507 1.209319

C -3.524103 0.811626 -0.000238

C -2.990108 1.243742 -1.209707

H -1.483933 2.469546 -2.144104

H -1.482497 2.467329 2.143963

H -3.428051 0.894562 2.135840

H -3.429501 0.896751 -2.136288

C 2.990110 1.243711 1.209726

C 1.909527 2.113001 1.215107

C 3.524103 0.811626 0.000245

C 2.989283 1.242538 -1.209299

C 1.908707 2.111818 -1.214788

C 1.384242 2.537951 0.000135

H 1.483935 2.469490 2.144158

H 3.429504 0.896695 2.136298

H 3.428048 0.894618 -2.135830

H 1.482494 2.467386 -2.143910

S 0.000000 3.620735 0.000043

O 0.000080 4.339553 -1.243844

O -0.000080 4.339519 1.243949

N 4.635876 -0.080695 0.000290

N -4.635876 -0.080695 -0.000307

C 5.930912 0.446779 -0.000520

C 7.045513 -0.401766 -0.000559

C 6.926892 -1.918341 0.000286

C 5.472074 -2.363184 0.001143

C 4.401964 -1.459137 0.001102

C -5.930912 0.446779 0.000471

C -7.045513 -0.401766 0.000497

C -6.926892 -1.918341 -0.000323

C -5.472074 -2.363184 -0.001135

C -4.401964 -1.459137 -0.001089

C 6.118325 1.836559 -0.001287

C 7.390973 2.382687 -0.002098

C 8.504757 1.552839 -0.002166

C 8.312835 0.179047 -0.001397

H 5.260068 2.494918 -0.001241

H 7.506217 3.459236 -0.002678

H 9.504995 1.965520 -0.002802

H 9.181252 -0.469962 -0.001444

C 7.624950 -2.479409 -1.249423

C 7.626028 -2.478097 1.249980

C 3.085920 -1.943635 0.001867

C 2.828513 -3.304346 0.002676

C 3.880159 -4.211607 0.002747

C 5.179647 3.726236 0.001979

H 2.257500 -1.248117 0.001816

H 1.802421 -3.650266 0.003252

H 3.694324 -5.277551 0.003381

H 6.001404 -4.433349 0.002026

C -6.118325 1.836559 0.001223

C -7.390973 2.382687 0.001999

C -8.504757 1.552839 0.002047

C -8.312835 0.179047 0.001298

C -3.085920 -1.943635 -0.001824

C -2.828513 -3.304346 -0.002603

C -3.880158 -4.211607 -0.002674

C -5.179647 -3.726236 -0.001941

C -7.624983 -2.479390 1.249377

C -7.625995 -2.478117 -1.250026

H -5.260068 2.494918 0.001194

H -7.506217 3.459236 0.002567

H -9.504995 1.965520 0.002655

H -9.181253 -0.469962 0.001332

H -2.257500 -1.248117 -0.001774

H -1.802421 -3.650266 -0.003155

H -3.694324 -5.277551 -0.003285

H -6.001404 -4.433349 -0.001991

H 7.153585 -2.099003 2.157934

H 8.679072 -2.191634 1.261630

H 7.575978 -3.568261 1.262336

H 8.677975 -2.192928 -1.262294

H 7.151703 -2.101292 -2.157365

H 7.574918 -3.569588 -1.260577

H -7.151761 -2.101257 2.157325

H -8.678008 -2.192910 1.262216

H -7.574950 -3.569568 1.260550

H -8.679038 -2.191652 -1.261708

H -7.575946 -3.568281 -1.262363

H -7.153528 -2.099038 -2.157973

The X, Y, Z coordinates of the DMAC-DPS molecule optimized in toluene applying the DFT/MN15 method. The coordinates X, Y, Z are given in angstroms.

C -2.581956 -1.379918 -0.013955

C -2.225785 -1.951103 -1.229364

C -2.080224 -1.855436 1.192355

C -1.198224 -2.925013 1.177091

C -0.831823 -3.503291 -0.034385

C -1.345655 -3.023744 -1.234189

H -2.649423 -1.566490 -2.148070

H -2.391715 -1.398311 2.1230630

H -0.790271 -3.325926 2.096411

H -1.050134 -3.499132 -2.161011

C -1.345655 3.023746 1.234184

C -2.225784 1.951106 1.229361

C -0.831822 3.50329 0.034379

C -1.198223 2.92501 -1.177095

C -2.080223 1.855433 -1.192357

C -2.581955 1.379918 0.013953

H -2.649423 1.566495 2.148068

H -1.050134 3.499137 2.161005

H -0.7902691 3.32592 -2.096417

H -2.391714 1.398306 -2.123064

S -3.674157 -0.000000 0.0000001

O -4.381533 0.010104 -1.247402

O -4.38153 -0.010104 1.247405

N 0.077360 4.600153 0.044742

N 0.077361 -4.600153 -0.044751

C -0.422738 5.900428 -0.084991

C 0.444747 6.99958 -0.084978

C 1.952945 6.859474 0.055589

C 2.366972 5.402217 0.1942

C 1.445788 4.347371 0.183103

C -0.422738 -5.900428 0.08499

C 0.444747 -6.999579 0.084983

C 1.952945 -6.859474 -0.055586

C 2.366972 -5.402217 -0.194199

C 1.445787 -4.347371 -0.183109

C -1.803297 6.106983 -0.213000

C -2.321822 7.383862 -0.343503

C -1.473057 8.482443 -0.348272

C -0.108930 8.271501 -0.218462

H -2.476266 5.260148 -0.208932

H -3.392178 7.514324 -0.440738

H -1.864383 9.486047 -0.449871

H 0.555095 9.128534 -0.220281

C 2.632983 7.459829 -1.185687

C 2.417518 7.635996 1.298495

C 1.902889 3.027994 0.309605

C 3.252405 2.752872 0.449319

C 4.176052 3.789105 0.464269

C 3.718028 5.091465 0.335826

H 1.193851 2.211184 0.29827

H 3.577237 1.724521 0.547441

H 5.233792 3.589098 0.573067

H 4.437968 5.902028 0.345266

C -1.803297 -6.106983 0.212999

C -2.321822 -7.383862 0.343507

C -1.473056 -8.482443 0.348282

C -0.108929 -8.2715 0.218472

C 1.902888 -3.027995 -0.309613

C 3.252404 -2.752872 -0.449325

C 4.176051 -3.789105 -0.46427

C 3.718028 -5.091465 -0.335824

C 2.632983 -7.459826 1.185691

C 2.417518 -7.635998 -1.29849

H -2.476267 -5.260149 0.208926

H -3.392178 -7.514324 0.440743

H -1.864382 -9.486046 0.449885

H 0.555096 -9.128533 0.220295

H 1.193850 -2.211185 -0.298282

H 3.577236 -1.724521 -0.547450

H 5.233792 -3.589098 -0.573067

H 4.437968 -5.902027 -0.34526

H 1.952208 7.234188 2.199999

H 2.152683 8.691379 1.213809

H 3.501264 7.57081 1.4100810

H 2.370211 8.513379 -1.296271

H 2.324180 6.930043 -2.088289

H 3.718956 7.392384 -1.099511

H 2.324181 -6.930038 2.088293

H 2.370212 -8.513376 1.296278

H 3.718957 -7.392381 1.099515

H 2.152683 -8.691381 -1.213802

H 3.501264 -7.570812 -1.410076

H 1.952208 -7.234192 -2.199995

The X, Y, Z coordinates of the DMAC-DPS dimer, that geometry was optimized in vacuum. The coordinates X, Y, Z are given in angstroms. The dimer geometry obtained from MC simulation was reoptimized applying the DFT/MN15method.

C 2.73691048008466 3.79134595576931 -0.42656262013388

C 1.56348646307512 4.53361954904218 -0.477792313300895

C 3.77712996674338 4.05687004959392 0.46000101917921

C 3.60923960508436 5.10444245766779 1.37039157765031

C 2.42949601517561 5.86752043324891 1.35791344388603

C 1.4159480600449 5.58915990900208 0.430058669277751

H 0.801291561120817 4.3044856218435 -1.21349784767139

H 4.69338471420524 3.47795101430688 0.437421152019889

H 4.38792552972638 5.34299211155145 2.08565422213931

H 0.517436149771242 6.19429629489804 0.415246286951885

C 4.03409746939617 -1.18114367379426 0.0231886070347692

C 4.06958133666811 0.0548323413126397 -0.633136248458223

C 2.8327993005711 -1.65085287805271 0.575464320641134

C 1.66532388175474 -0.874601502793035 0.492050056872771

C 1.68722401810079 0.365051314587247 -0.153852407283508

C 2.8937732937813 0.793754233287635 -0.698724653963965

H 4.9703731069343 0.433677742600557 -1.10299822628651

H 4.92621193241395 -1.79270892860451 0.0940910618609552

H 0.745517220913974 -1.24820545017792 0.92646156792812

H 0.783736847769525 0.958502300257208 -0.236332794389457

S 2.94140942884567 2.40126864163646 -1.67362751922648

O 1.59885346435739 2.44628778222272 -2.62845056947496

O 4.4487169020141 2.49188880258377 -2.32486130506417

N 2.7856600746792 -2.94066414294431 1.21123139101365

N 2.26495089881343 6.94192615839345 2.30165723527223

C 2.62808000006506 -4.099455782281 0.408940275202815

C 2.51591669027341 -5.37598108522781 1.00624256877629

C 2.5411374465502 -5.59002189452654 2.52868382075108

C 2.74494334673136 -4.26520082105714 3.28188323073274

C 2.86358580907641 -3.01994477700746 2.6241993438612

C 2.79850130998345 8.21951650925397 1.99623300594455

C 2.68314129101034 9.28495397598007 2.91754837826544

C 1.98172295934794 9.13869166925551 4.27803496229358

C 1.42039096151155 7.72064471770858 4.47446345392975

C 1.57512502199562 6.69507166528967 3.51342755573069

C 2.5760465624232 -3.98074981500608 -0.996987418070384

C 2.42932904309881 -5.10615435453588 -1.80667516271212

C 2.32037774220201 -6.37494470729028 -1.22950754793867

C 2.36332359241377 -6.48756700093032 0.161503130760396

H 2.65806801295232 -3.00638459891312 -1.4597365575609

H 2.41234001579883 -4.98767874860306 -2.88585851933288

H 2.20804180826666 -7.2591913220444 -1.84733801970803

H 2.27574217341423 -7.47322900639874 0.606125997815173

C 1.18873582691776 -6.22683279944642 2.97306232118137

C 3.70886921588746 -6.55998565056903 2.88629752524438

C 3.07089391278383 -1.84692709684558 3.38169312885939

C 3.14408257592462 -1.89596784831156 4.77217124872799

C 3.01551287122775 -3.11852244279938 5.43648568452694

C 2.82178094028844 -4.27825840525478 4.68416952434482

H 3.17840027397406 -0.894417675047055 2.87968061817696

H 3.30388886727851 -0.97894389200414 5.33032331170276

H 3.0687020994102 -3.17093965283403 6.51856826456579

H 2.72728909666465 -5.22613473047874 5.20346453200087

C 3.44519494422212 8.43186942888602 0.759676176392047

C 3.98279273589703 9.67622739102302 0.43785298378076

C 3.88412484340539 10.7364066173637 1.3428606230104

C 3.23735745907267 10.5255262405873 2.56160966293415

C 1.04192227809917 5.41254667758905 3.77056075019968

C 0.361360225163037 5.14414707581046 4.95690670546882

C 0.199625445831843 6.14903866406834 5.9141964180372

C 0.730219518980388 7.41477988845831 5.65875357917025

C 3.00720140723314 9.44007673578947 5.41374353944852

C 0.810449339691686 10.1650815366171 4.35880720612063

H 3.52489608377013 7.62181141297951 0.0468660031387788

H 4.47523129935972 9.81188752037613 -0.519638148053212

H 4.29995571638147 11.7096262578442 1.10544330797249

H 3.16048431829236 11.3521883467474 3.26018095561978

H 1.16130427384734 4.62264166957801 3.04110736848207

H -0.0360785000014097 4.14859316092981 5.12759692289342

H -0.326126478227752 5.95335052269061 6.84255263584843

H 0.606104646893083 8.19150559200332 6.4060709281782

H 4.67003112432243 -6.13200947007219 2.58349666322945

H 3.58761449261345 -7.52300346409185 2.37998610726976

H 3.74313399334421 -6.75102118561235 3.96369890655663

H 1.0257983606032 -7.1871035256542 2.4730238737072

H 0.349177976581402 -5.57042736894791 2.72379870615571

H 1.17858607077676 -6.40340493619744 4.05395706035279

H 3.83964465948011 8.72983718952804 5.37733273286795

H 3.41522785132271 10.4512345605939 5.31317292489681

H 2.53423205016327 9.36356207925124 6.39847159454736

H 0.0774217685341198 9.97295861582274 3.5685916799676

H 1.17856526398799 11.1894987078326 4.24248691755377

H 0.298508651809447 10.1008995034535 5.32450632852711

C -2.65843773380954 -0.308599190355816 -4.2335520783215

C -3.84859757278522 0.123139436995805 -3.65782831636014

C -1.42081011236014 0.257076731280668 -3.95117309393492

C -1.36172108633889 1.28161978208183 -2.99757032421636

C -2.54129107504628 1.71494478832258 -2.36886718187626

C -3.78056822336588 1.15065138623653 -2.71190210248344

H -4.79420763892514 -0.317238505892478 -3.95282870949096

H -0.529993350179185 -0.083251590055485 -4.46687442619513

H -0.403795978299462 1.7323385202003 -2.75273694372354

H -4.68023604212195 1.51607657642864 -2.23009751099769

C -1.38004544225405 -4.81245831280317 -3.01745230782269

C -1.41594775005121 -3.68745231990996 -3.84825165599842

C -2.56556371477013 -5.50370285797412 -2.71999488357025

C -3.78982962885349 -5.0781518128119 -3.25761199445306

C -3.83963962409096 -3.94813283709977 -4.08229854381648

C -2.64992288456893 -3.27885498494153 -4.3439191253664

H -0.509488433821286 -3.15693912808736 -4.11654321092921

H -0.441975425680214 -5.15943636172752 -2.59836277320771

H -4.69166886703371 -5.63474238348069 -3.02947140407266

H -4.76440948591113 -3.60220386371447 -4.53021431998394

S -2.72241627721689 -1.74932394781665 -5.43994324431365

O -4.21401698810395 -1.75019457363085 -6.13662207463141

O -1.33269296780627 -1.70415848716334 -6.3183476651585

N -2.52016095774736 -6.66096283418659 -1.86381904888532

N -2.50328240413099 2.71171440122603 -1.32674724683397

C -2.27886332170714 -7.93435157501744 -2.43667953616703

C -2.18016076876912 -9.08315087504246 -1.61929598688582

C -2.31740472206294 -9.03093726761242 -0.0887621789140956

C -2.60327996385568 -7.60302025197752 0.404545140464278

C -2.68847836962402 -6.49405102456776 -0.468446994140455

C -2.29030103727911 4.08374798883278 -1.63672390450817

C -2.48877969901587 5.05364557878419 -0.621951119254931

C -3.1351091079904 4.6177483011114 0.703249785441438

C -2.61449150879587 3.21825957063799 1.06447503470409

C -2.4177995736071 2.27878534847692 0.0227680392055735

C -2.13801919923262 -8.05892840807761 -3.8358180469654

C -1.89987220066748 -9.29938190581377 -4.42358828407046

C -1.79757966989971 -10.4432938073347 -3.62739350365495

C -1.93905556170203 -10.3175560225607 -2.24449047097194

H -2.21616112979022 -7.18239580616841 -4.46520162556993

H -1.79604240881728 -9.36633379877109 -5.50177487015646

H -1.61208313590761 -11.4149228198632 -4.07239983076145

H -1.85854037182693 -11.2080400709488 -1.62968201097268

C -3.48465474521675 -9.96645167984017 0.350975554187784

C -0.987320403043197 -9.53036936660595 0.555167359179715

C -2.9435145481413 -5.20971546351418 0.05981327992857

C -3.1125103107739 -5.01843227553361 1.42939388952197

C -3.03026393258794 -6.10564130898002 2.30389417724565

C -2.77814018087132 -7.3747437368161 1.77908501712847

H -3.00985842635364 -4.35932399666132 -0.605733909071278

H -3.31105747944608 -4.02055409427273 1.80785264776749

H -3.16242495046162 -5.97124155981762 3.37208678797916

H -2.71429346642574 -8.21652782095902 2.46070758341803

C -1.87499454028956 4.48395132311016 -2.92059244946226

C -1.61689545114738 5.82855024019367 -3.19313770056903

C -1.76098964474874 6.78933169594674 -2.1894224203195

C -2.19919734416284 6.39369425300369 -0.920634861501365

C -2.11334352179105 0.938088660676785 0.333888260514535

C -1.97441127793983 0.534416120317025 1.66399976685074

C -2.11316115883067 1.46368261784289 2.69765685256669

C -2.43245774129612 2.79024280005983 2.38703428871076

C -2.89445813422113 5.63562158483279 1.83839760026739

C -4.67982771517828 4.51193387637017 0.470309962864388

H -1.74112826542906 3.74891952142542 -3.7026669587055

H -1.29265257739286 6.11557482903757 -4.18787323698481

H -1.5531740708067 7.83467286740199 -2.39121638977274

H -2.34085962261999 7.1519944765915 -0.160011844864992

H -1.98987825354471 0.214469812758654 -0.461851418933771

H -1.75595224161843 -0.505756337518395 1.88693651143571

H -1.99500552634081 1.16032487362713 3.73248574223892

H -2.55996945301249 3.49759665131266 3.19722113949887

H -1.05770865313883 -9.52930981813681 1.648187362469

H -0.156376292061378 -8.87949910413032 0.262823194075397

H -0.755095206114289 -10.550738825372 0.232509940514217

H -3.3019405730306 -10.9977923948193 0.0324062851071006

H -4.4291311904816 -9.63371162175065 -0.0915009631828705

H -3.59793864433097 -9.96758842717088 1.439970331268

H -1.83051516492144 5.74331105545815 2.07467847808904

H -3.29457595839065 6.61740870132479 1.57016582084674

H -3.41780953291715 5.32783332031528 2.74837273214696

H -5.08508396633805 5.49083041326733 0.189663120066055

H -5.17658929567094 4.17178276022971 1.38629291063188

H -4.90965856283236 3.80298744201034 -0.330950677102464
